# The CLOSED protocol to assess optic nerve sheath diameter using color-Doppler: a comparison study in a cohort of idiopathic normal pressure hydrocephalus patients

**DOI:** 10.1186/s13089-022-00291-5

**Published:** 2022-10-29

**Authors:** Raffaele Aspide, Giacomo Bertolini, Laura Maria Beatrice Belotti, Luca Albini Riccioli, Francesco Toni, Diego Mazzatenta, Giorgio Palandri, Luigi Vetrugno, Daniele Guerino Biasucci

**Affiliations:** 1 IRCCS Istituto delle Scienze Neurologiche di Bologna, Anesthesia and Neurointensive Care Unit, Bologna, Italy; 2 Department of Biomedical and Neuromotor Sciences, Department of Neurosurgery, University of Bologna, Bologna, Italy; 3 IRCCS Istituto Delle Scienze Neurologiche Di Bologna, Epidemiology and Statistic Unit, Bologna, Italy; 4 IRCCS Istituto Delle Scienze Neurologiche Di Bologna, Neuroradiology Unit, Bologna, Italy; 5 IRCCS Istituto Delle Scienze Neurologiche Di Bologna, Department of Neurosurgery, Bologna, Italy; 6 Department of Medical, Oral, and Biotechnological Sciences, University of Chieti-Pescara, Chieti, Italy; 7 “Tor Vergata” University of Rome, Department of Clinical Science and Translational Medicine, Rome, Italy

**Keywords:** Optic nerve sheath diameter, Hydrocephalus, Point-of-care ultrasound, Intracranial pressure, Neurocritical care

## Abstract

**Background:**

Sonographic assessment of the optic nerve sheath diameter represents a promising non-invasive technique for estimation of the intracranial pressure. A wide inter-observer variability, along with a lack of a standardized protocol for the optic nerve sheath diameter measurements, could lead to over- or under-estimation. The present study was aimed at evaluating feasibility of color-Doppler for better delineating optic nerve sheath borders, comparing it to B-mode imaging, using the magnetic resonance measurements as a comparison.

**Methods:**

Optic nerve sheath diameters were evaluated using magnetic resonance by an expert radiologist in a cohort of patients with suspected idiopathic normal pressure hydrocephalus. Magnetic resonance findings were evaluated twice. In the first half of this cohort, optic nerve sheath diameters were measured using B-mode only, in the second half applying color-Doppler. Measurements obtained using these two techniques were compared to magnetic resonance imaging measurements. The Bland–Altman analysis and concordance correlation coefficient were computed to quantify the strength of agreement between the two magnetic resonance assessments. Box plots and average (± SD) were used to compare assessments by sonographic and magnetic resonance methods.

**Results:**

Fifty patients were included. MRI assessment showed a moderate concordance correlation coefficient. Optic nerve sheath diameters measured applying color-Doppler were lower (*p* < 0.001) and less scattered compared to B-mode assessment, which approached more to magnetic resonance measurements.

**Conclusions:**

In this cohort of patients, magnetic resonance showed high intra-rater variability in optic nerve sheath diameter assessments. Optic nerve sheath diameter assessments using color-Doppler yielded lower and less scattered diameters compared to B-mode only.

## Introduction

The optic nerve sheath diameter (ONSD) measurement has been reported as a tool for the non-invasive assessment of intracranial pressure (ICP) [1–4]. The explanation for this use is based on anatomical continuity of the optic nerve (ON) sheath with the intracranial dura mater and cerebrospinal fluid movement according to pressure gradients. ICP variations are detectable measuring the diameter of the ON sheath which varies with thickening of the subarachnoid spaces [1]. The increase in ONSD occurs a few seconds after the increase in ICP and can be correctly detected even with ultrasound (US) [2, 5].

Physiological ONSD values of post-mortem human adults measure 4 mm [3], while the ON without sheath has a diameter of about 3 mm [4]. In healthy subjects, the optic nerve sheath instead has an average thickness of about 0.4 mm and the subarachnoid space between the nerve and the sheath measures approximately 0.1 mm [4, 6].

Recently, Ertl et al. studied in 187 healthy volunteers a range of physiological ONSD between 4.9 e 5.3 mm assessed by US [7].

Despite many methodological comparison studies of non-invasive methods, a validated gold standard for the ONSD assessment is unavailable. Nevertheless, recent evidence confirmed that a high-resolution magnetic resonance image (MRI) allows measuring ONSD accurately [8–10]. In a dedicated study, focusing on children and adolescents, using both MRI and B-mode US, a good reproducibility and repeatability of the ONSD measurement, with a repeatability coefficient between 0.34 and 0.46 mm, was described [11]. Conversely, other papers have disputed those findings because of a lacking standardized ONSD sonographic methodology [12]. Our group previously presented a dedicated bundle [13], based on US mode and the identification of some anatomical landmarks also using color Doppler, to improve the reliability and standardize the ONSD assessment. This so-called CLOSED protocol has recently been applied by other authors (Pansell et al.) with promising results [14]. Trying to shed some light about this debated topic, the following study has been designed. More precise indications regarding the landmarks to be used for the measurement with US come from a recent systematic review of 63 articles [15].

The present study hypothesizes that ONSD assessment using a US standard bundle implemented by color Doppler may yield a more accurate measurement than simple B-mode imaging. The aim of the present study was to evaluate feasibility of ONSD sonographic assessment applying color-Doppler and using B-mode only comparing them with MRI ONSD assessment.

## Materials and methods

### Study design, participants and setting

A single-center cross-sectional cohort study was conducted between February 2018 and April 2019. The authors selected an idiopathic Normal Pressure Hydrocephalus (iNPH) population for this study, such as a homogeneous consecutive cohort without intracranial hypertension. Participants had to be I) older than 18 years and II) with suspected iNPH diagnosis after a multidisciplinary evaluation (PRO-Hydro) based on clinical and radiological findings [16]. Patients with ON diseases, the presence of a central nervous system mass, other primary causes of possible altered ICP and those who declined participation in the present study, were excluded. Eligible patients underwent a specific 3-Tesla brain MRI, using a protocol described below, and an immediate subsequent ONSD sonographic assessment. The study was conducted in accordance with the declaration of Helsinki and the principles of Good Clinical Practice. The study protocol was approved by the local ethics committee (Cod. CE 17115). All patients gave their written informed consent to participate. We used Strengthening the Reporting of Observational Studies in Epidemiology (STROBE) guidelines for cohort studies as reporting guidelines [17].

### Bias

The same MRI and US equipment were used to limit a systematic error in ONSD measurements, and the two measurements were obtained in rapid sequence to minimize ICP orthostatic changes. Same neuroradiologist and neurosonologists performed all measurements. Patients with iNPH require a complex process of differential diagnosis, therefore the population that can be recruited for the duration of the study, albeit in a reference center, is small.

### Demographic and clinical variables

The age and gender of patients were recorded for each enrollment. iNPH was suspected according to criteria established by Relkin et al. [18].

### MRI variables

A 3-Tesla whole-body scanner MRI (Magnetom Skyra, Siemens Healthcare, Erlangen, Germany) used a 32-channel phased-array head coil. The neuroradiologist selected the best sequence available within the specific protocol, using a sagittal T1-weighted rapid three-dimensional gradient echo technique (Repetition Time, 2300 ms; Echo Time, 2.98 ms; flip angle, 9°; thickness, 1 mm; 160 slices; field of view, 256 × 248 mm; matrix, 256 × 248: 1 mm × 1 mm) which showed contrasts between endo-orbital fat and sheath providing optimal morphological imaging for ONSD evaluation. The measurements were performed on reconstructions carried out parallel and orthogonal to the ON. The assessments were performed twice, blindly, on the same images, at 2 weeks from each other.

### Ultrasound variables

**Fig.1:**
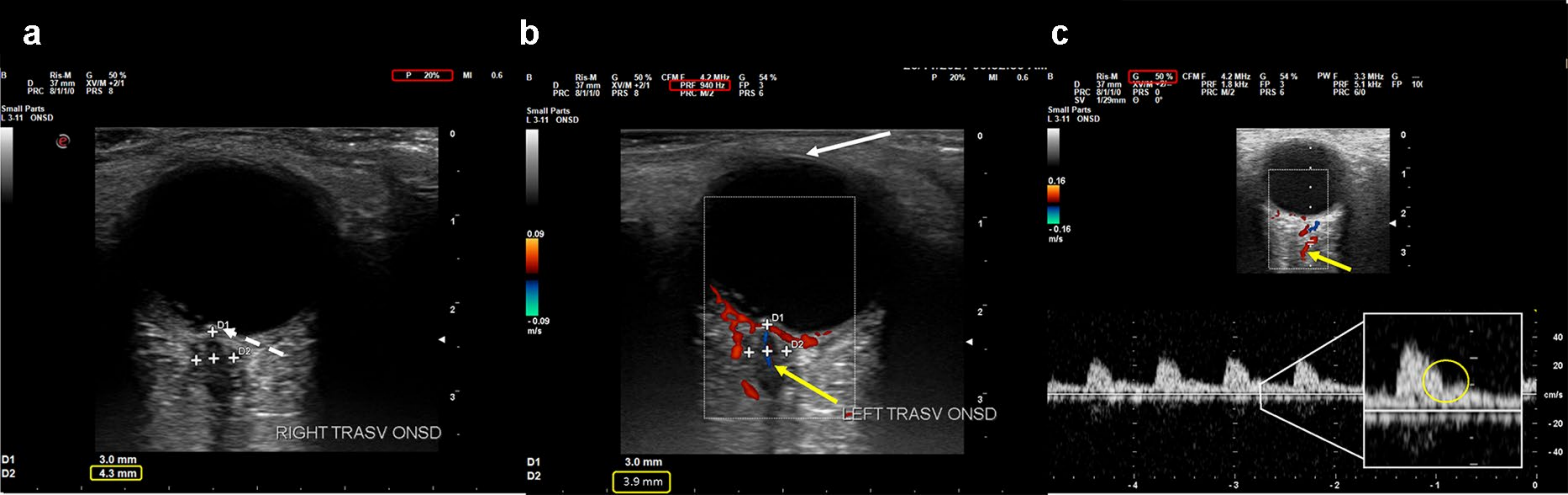
Three ultrasound images of the eyeball and optic nerve with sheath, enriched by the parameters/landmarks highlighted in CLOSED protocol. **a** B-mode image in gray-scale: dashed white arrow points to the optic disc, in yellow window 4.3 mm ONSD measurement, in red window acoustic power output = 20%; **b** same image but with color-mode: white arrow points to the lens, in yellow window 3.9 mm ONSD measurement, in red window PRF 940 Hz (almost 1 Kh), yellow arrow points to central retinal artery; **c** same image but with color-mode plus Doppler wave (at the bottom): in red window Gain 50%, yellow arrow points to ophthalmic artery, in the enlarged panel the typical nock of the ophthalmic artery flow

US settings and ONSD evaluation were performed according to the CLOSED protocol, a bundle which includes safety measures and technical procedures already reported in a previous paper [13]. US was performed disjunctively in B-mode and color-Doppler mode, using a MyLab^™^Twice US system (Esaote, Italy) equipped with a 11–3 MHz linear transducer (Fig. 1).

### Data variables

ONSD was measured 3 mm behind the optic disc on MRI and US imaging. All measurements were performed bilaterally and on the two planes (transverse and sagittal), named right and left transverse diameter (TDR and TDL, respectively) and right and left sagittal diameter (SDR and SDL, respectively). All the investigators were blinded to the other measurements. One experienced neuroradiologist (FT) performed ONSD measurements two times on MRI, to determine the intra-agreement. Sonographic ONSD assessments were performed by one expert neurosonologist (RA) supported by a further expert physician proficient in neurosonology (GB).

### Statistical analysis

Demographical variables were compared between US subgroups (US B-mode group vs. US color-Doppler mode group): Mann–Whitney *U* test and Chi-square test were used to compare age and sex, respectively.

The evaluation of intra-reliability of ONSD assessment using MRI was performed calculating Bland–Altman plots [19]: the agreement between the two-consecutive assessment of the same neuroradiologist (intra-observer) were quantified by plotting the difference between measurements against their mean and by constructing limits of agreement. Mean and the standard deviations of the differences between two measurements were used for statistical limits; they represent the maximum deviation expected for the 95% of the differences between the two measurements. In addition, the Lin’s Concordance Correlation Coefficient (CCC) [20] with 95% confidence intervals (CI) was computed to quantify the strength of intra-observer agreement: this coefficient provided indications on a standardized relationship scale about precision (how close the data are about the line of best fit) and accuracy (how far the line of best fit is from the 45-degree line through the origin, which represents perfect agreement). Lin’s CCC values < 0.20 were considered as “poor” while values > 0.80 were considered as “excellent”, values between > 0.20 and < 0.80 were considered “moderate”.

The two different US methods (B-mode group and color-Doppler mode) were described using median and interquartile (IQR) range; differences in distribution were graphically displayed by box-plot and compared using Mann–Whitney *U* test. Then, differences between each of the two US methods and MRI were described by box-plot and evaluated by calculating average differences and their standard deviations.

Statistical analysis was performed using Stata statistical software version 14 (Stata Corp LLC, College Station, 101 TX, USA).

## Results

Fifty consecutive patients, 29 males and 21 females, fulfilled the eligibility criteria with a mean age [± standard deviation (SD)] of 76 ± 8 years. MRI was performed in all 50 patients. In 27 patients ONSD assessment was performed using US B-mode, while in 23 using US color-Doppler mode. No differences in demographic variables were observed in the two US subgroups analyzed (Table 1).

**Table 1:** Demographic characteristics and statistical comparison according to the ultrasound modality

	Ultrasound B-mode (*n* = 27)	Ultrasound color-Doppler mode (*n* = 23)	*p*-value
Age			
Median (IQR)	75 (74–79)	78 (74–82)	0.11
Sex			
Male, *n* (%)	18 (62)	11 (38)	0.18
Female *n* (%)	9 (43)	12 (57)	

There are no demographic differences between the two subgroups

*IQR* interquartile range

### MRI variables—intra-rater agreement

**Fig. 2:**
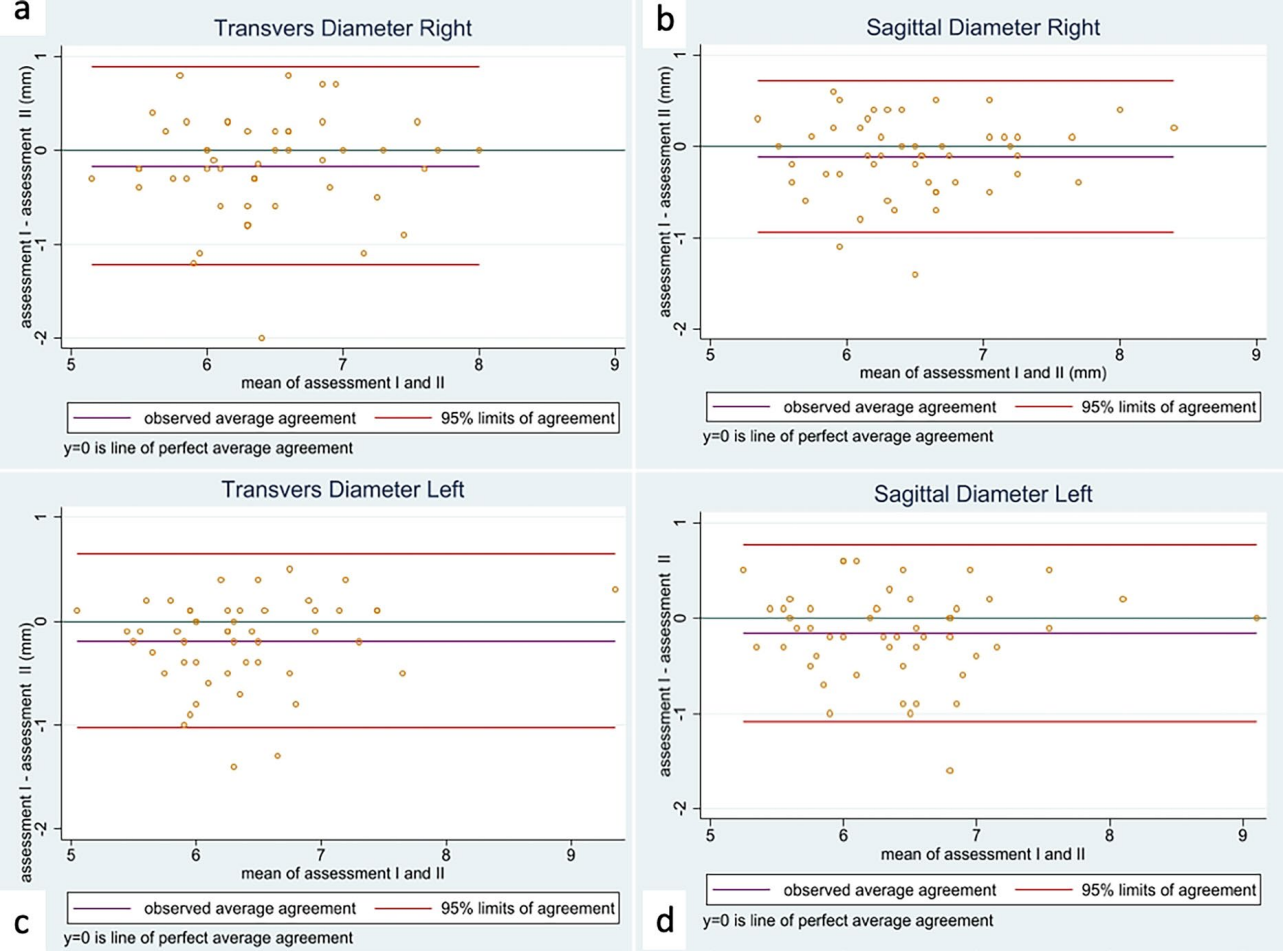
Bland–Altman plots displaying the intra-rater agreement for neuroradiologist with two sets of measurement in magnetic resonance imaging. The values in the reported axes are in millimeters. **a** Average difference (violet line): − 0.165; 95% limits of agreement (red lines): − 1.216, 0.886. **b** Average difference (violet line): − 0.112; 95% limits of agreement (red lines): − 0.944, 0.721. **c** Average difference (violet line): − 0.190; 95% limits of agreement (red lines): − 1.022, 0.642. **d** Average difference (violet line): 0.154; 95% limits of agreement (red lines): − 1.080, 0.772

For each of the two different diameters for each eye, first and second assessments were compared. Table 2 shows intra-rater reliability using Lin’s CCC: the neuroradiologist only in TDL has an excellent CCC: 0.808. Bland–Altman plots show the difference of the two assessments by the neuroradiologist against their mean for each diameter (Fig. 2).

**Table 2:** Intra-rater agreement in MRI assessment of the neuroradiologist

NRD—assessment I vs. II	TDR	SDR	TDL	SDL
CCC (95% CI)	0.678 (0.530, 0.827)	0.796 (0.693, 0.898)	**0.808** (0.715, 0.902)	0.791 (0.688, 0.895)

A CCC (95% CI) < 0.20 was considered as “poor” and > 0.80 was considered as “excellent”. Comparison between the assessment I and II of the neuroradiologist

*NRD* neuroradiologist, *CCC* Lin’s concordance correlation coefficient, *TDR* transverse diameter right, *SDR*, sagittal diameter right, *TDL*, transverse diameter left, *SDL*, sagittal diameter left

### Two different sonographic modalities (B-mode and color-Doppler mode) versus MRI

**Fig. 3:**
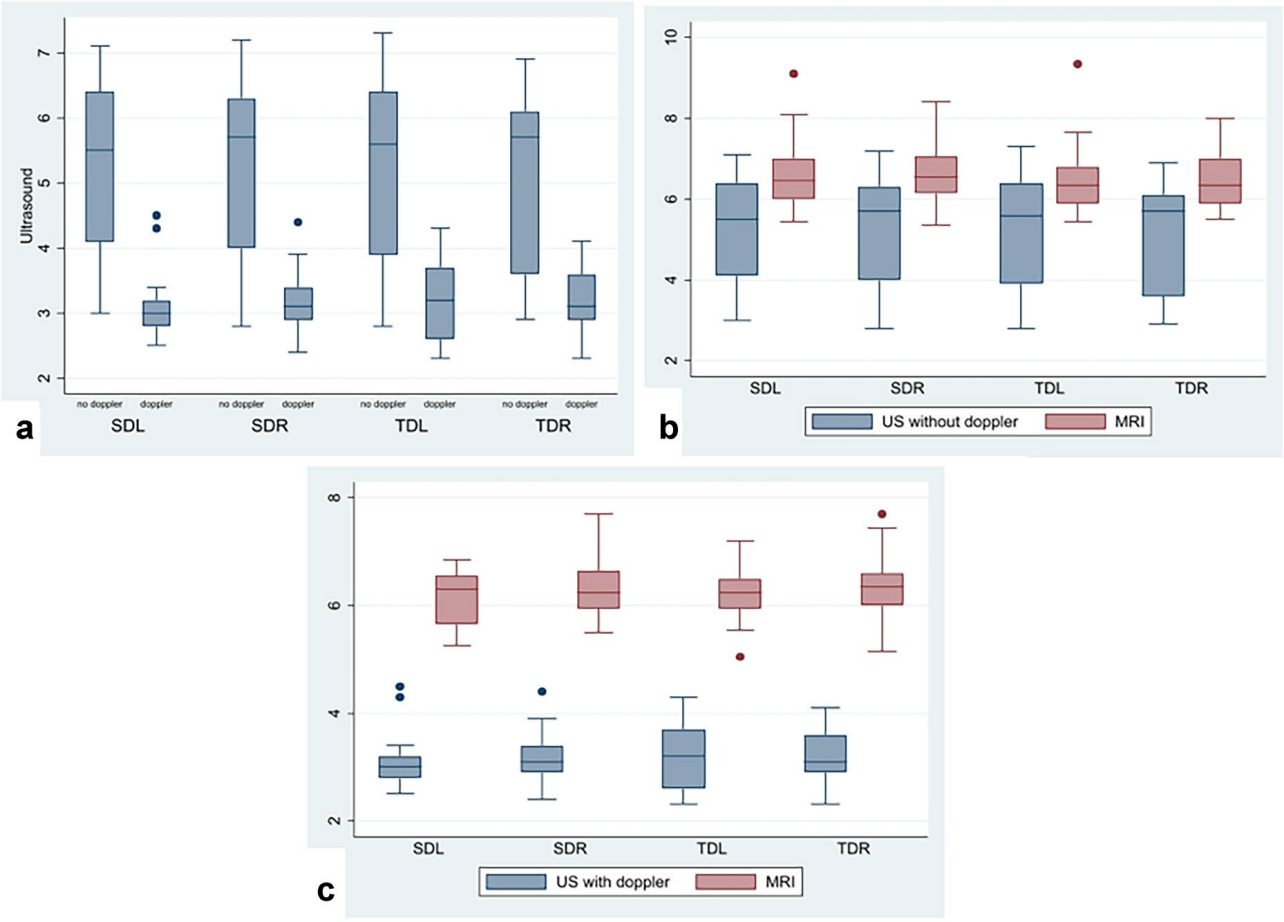
The values in the reported axes are in millimeters. **a** Color-Doppler measurements are less scattered and significantly lower than those in B-mode. **b** MRI vs B-mode (without Doppler) in 27 patients; **c** MRI vs color-Doppler mode in 23 patients; box plots show a clear difference in comparison between the MRI and the two US modalities, highlighting an evident difference in all the analyzed diameters. *SDL* sagittal diameter left, *SDR* sagittal diameter right, *TDL* transverse diameter left, *TDR* transverse diameter right

There are substantial and statistically significant differences between measurements made by the two different ultrasound methods: for all four diameters, median values statistically differ (*p* < 0.001) (Table 3). Moreover, color-Doppler measurements are less scattered and significantly lower than those in B-mode (Fig. 3a). Finally, MRI measurements were compared to B-mode (27 patients) and to color-Doppler (23 patients). Box-plot shows the distribution of MRI measurements compared to US B-mode (Fig. 3b) and US color Doppler (Fig. 3c): ONSD measurements by US were always lower than those by MRI. Main differences between US color Doppler and MRI compared to US B-mode are reported in Table 4.

**Table 3:** Median and IQR of color-Doppler and B-mode US ONSD assessment: for all four diameters the comparison between median values differs significantly (*p*-value < 0.001)

		US color-Doppler (*n* = 27)	US B-mode (*n* = 23)	*p*- value
TDR	Median (IQR)	3.1 (2.9–3.6)	5.7 (3.6–6.1)	** < 0.001**
SDR	Median (IQR)	3.1 (2.9–3.4)	5.7 (4–6.3)	** < 0.001**
TDL	Median (IQR)	3.2 (2.6–3.7)	5.6 (3.9–6.4)	** < 0.001**
SDL	Median (IQR)	3 (2.8–3.2)	5.5 (4.1–6.4)	** < 0.001**

*IQR* interquartile range, *US* ultrasound, *TDR* transverse diameter right, *SDR* sagittal diameter right, *TDL* transverse diameter left, *SDL*, sagittal diameter left

**Table 4:** Comparison between MRI and two different US methods in ONSD assessment

Difference MRI vs. US B-mode (27 pt.)	TDR	SDR	TDL	SDL
Average (SD)	1.338 (1.629)	1.416 (1.574)	1.274 (1.681)	1.335 (1.546)

Difference MRI vs. US color-Doppler (23 pt.)	TDR	SDR	TDL	SDL
Average (SD)	3.178 (0.769)	3.176 (0.500)	3.011 (0.780)	3.052 (0.563)

The average difference between MRI and US B-mode measurements has much lower values than the average difference between MRI and US color-Doppler mode for each diameter

*MRI* magnetic resonance imaging, *US* ultrasound, *SD* standard deviation, *TDR*, transverse diameter right, *SDR*, sagittal diameter right, *TDL* transverse diameter left, *SDL* sagittal diameter left

## Discussion

Our analysis showed poor accuracy of ONSD measurements by MRI with moderate intra-rater variability. Our previous proof-of-concept study [13] suggested a possible discrepancy between B-mode and color-Doppler US measurements. The comparison results between MRI and US methods are reported in Table 4 and Fig. 3b and c. Our data showed poor agreement between MRI and US regardless of the ultrasound modality. However, color-Doppler based measurements appear less scattered and significantly lower than using B-mode only and MRI (Fig. 3b, c).

B-mode and MRI measurements were substantially higher than clinically expected (> 5 mm). On the other hand, measurements yielded by color-Doppler application were lower and more reliable than clinically expected.

Other studies compared ONSD values of US assessment with MRI assessment. Steinborn et al. [21] demonstrated moderate CCC agreement in a pediatric population. However, the results were obtained using an ultra-high frequency (17 MHz) linear array transducer and compared to a heavily turbo spin echo sequence T2-weighted. Likewise, Bäuerle et al. [22] found an agreement between US and MRI in 15 healthy volunteers, but also this discrepancy could be attributed to the use of non-standard resolution MRI sequences. Moreover, even Patterson et al. [23] reported an ONSD agreement in a cohort of intracranial hypertension patients. However, in a more recent paper, Raval et al. [12] did not find significant agreement in transverse, inferior or sagittal US views and MRI in the axial view of ONSD, raising concerns about this association because of different US ocular plane orientation, different image quality and different angle of transection.

Unfortunately, the lack of agreement is a recurrent element in US assessment, partially due to the intrinsic user- and technique-dependence of those findings. Despite ONSD measurement, as non-invasive estimator of intracranial pressure, is widely discussed by numerous prospective studies and meta-analysis [24–27], very few studies discuss how to properly interpret a different echogenicity between confining structures and correctly identify the real margin of the ONSD.

**Fig. 4:**
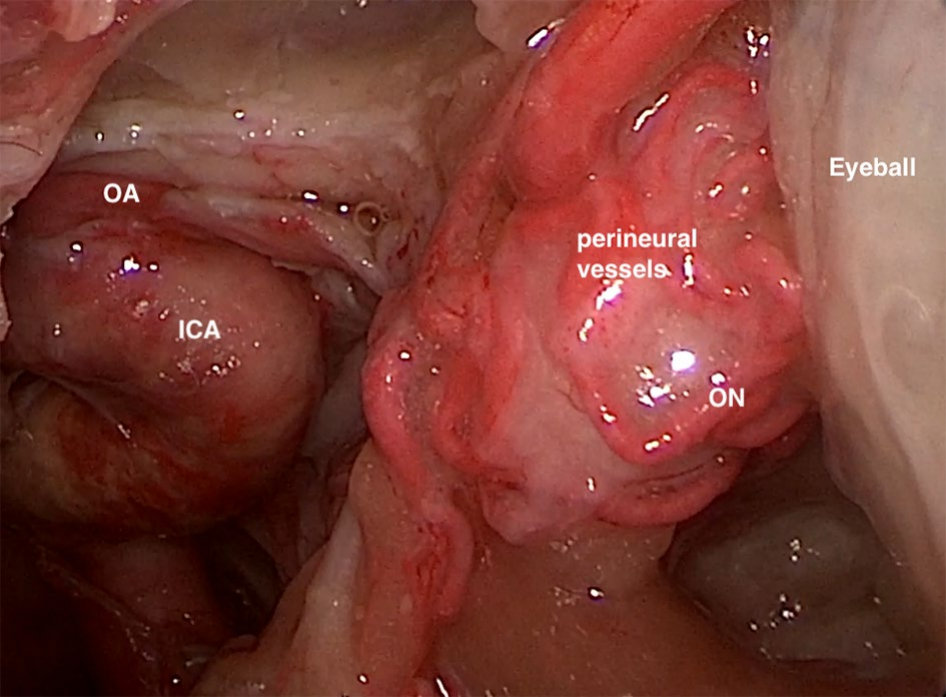
Cadaver endoscopic anatomical exemplificative view. It is possible to appreciate how in the distal segment of the optic nerve (ON), contiguous to the posterior portion of the eyeball, the perineural vessels surround the sheath together with the fat. Instead, in the proximal segment, the optic nerve is contiguous to the ophthalmic artery (OA), which runs parallel to the internal carotid artery (ICA)

As proposed in the CLOSED protocol [13], we defined a technique based on standardized setting and identification of some new anatomical landmarks even adding color-Doppler (central retinal artery, central retinal vein and ophthalmic artery) in order to obtain more accurate and reliable images and consequent measurements (Fig. 1). Interestingly, performing a measurement comparison between the US B-mode on gray-scale and color-Doppler mode, we noticed that the measurements performed with MRI and US B-mode are more similar to each other, compared to those performed with US color-Doppler (Table 4). Considering a comparison to the neuroanatomical post-mortem reference [3], a possible explanation for these findings could be that MRI such as B-mode ultrasound could overestimate the diameter, since a clear distinction between the nerve sheath and perineural vessels is not easily identifiable. At the same time the color-Doppler US could underestimate the diameter itself for excessive representation of the vessels (Fig. 4). MRI without and with contrast enhancement does not clearly display perineural vessels [28]. US color-Doppler seems to discriminate better, using perineural vascular components as landmark structures, leading to a more accurate measurement of the ONSD. The CLOSED protocol, with its simple indications on settings, image quality and implementation of color-Doppler mode, could reduce operator-dependency of the ONSD measurements, as Pansell et al. demonstrated by finding an excellent inter- and intra-rater reliability and low risk of inter-rater bias. Pansell also added an anthropomorphic correction, as eyeball diameter and ON diameter, that can represent a further implementation of the protocol mentioned above. [14]

### Limitations

First of all, the two sonographic methods (B-mode and color-Doppler US) were not assessed in the same group of patients. Therefore, a direct correlation between the US modality and the differences in the ONSD measurements could not be confirmed. Finally, our results cannot be generalized due to the small sample size and iNPH population studied, which could be highly selective.

## Conclusions

Our data suggest that application of color-Doppler using a standardized safety bundle may allow a more accurate assessment of ONSD than the B-mode only. Surprisingly, MRI did not reach enough accuracy and reproducibility for ONSD diameter measurements. This may be because the application of color-Doppler allows better delineating ON sheath border, yielding more reliable and accurate measurements. These findings offer an interesting scenario that might warrant further investigation.

## Data Availability

The data that support the findings of this study are available from the corresponding author, RA, upon reasonable request.

## Code availability

A specific custom code will be given by the corresponding author, RA, upon reasonable request.
